# The Portrayal of Natural Environment in the Evolution of the Ecological Public Health Paradigm

**DOI:** 10.3390/ijerph110101005

**Published:** 2014-01-10

**Authors:** Christopher Coutts, Annet Forkink, Jocelyn Weiner

**Affiliations:** 1 Department of Urban and Regional Planning, Center for Demography and Population Health, Florida State University, 113 Collegiate Way, Tallahassee, FL 32306, USA; E-Mail: jw11f@my.fsu.edu; 2 Department of Urban and Regional Planning, Florida State University, 113 Collegiate Way, Tallahassee, FL 32306, USA; E-Mail: af11g@my.fsu.edu

**Keywords:** health, nature, natural environment, green space, green infrastructure, urban planning, built environment, ecology

## Abstract

This paper explores the conceptualization of the natural environment in an evolving ecological public health paradigm. The natural environment has long been recognized as essential to supporting life, health, and wellbeing. Our understanding of the relationship between the natural environment and health has steadily evolved from one of an undynamic environment to a more sophisticated understanding of ecological interactions. This evolution is reflected in a number of ecological public health models which demonstrate the many external and overlapping determinants of human health. Six models are presented here to demonstrate this evolution, each model reflecting an increasingly ecological appreciation for the fundamental role of the natural environment in supporting human health. We conclude that after decades of public health’s acceptance of the ecological paradigm, we are only now beginning to assemble knowledge of sophisticated ecological interdependencies and apply this knowledge to the conceptualization and study of the relationship between the natural environment and the determinants of human health.

## 1. Introduction

The recognition that human health is affected by a complex web of environmental determinants and processes, including the reciprocal determinism between the environment and human actions, represents an ecological way of thinking about health. Until somewhat recently, our ecological models have not fully reflected the complex ecological nature of humans as inseparable from the natural environment (the physical world and everything in it not made by people) and human health as a product of this interdependence. A number of contemporary ecological models present other ecological spheres (e.g., the built environment, community, and economy) as dependent upon the natural environment and biosphere that are essential to delivering health-supporting services. These services range from the provision of the ecosystem services of food, water, and air, to more nuanced stress-reducing and social capital services, to the role of forests in mitigating the health threats posed by climate change [1,2]. 

The purpose of this paper is to outline the acceptance of the natural environment in our evolving ecological public health paradigm, a paradigm that has steadily incorporated the complex reciprocity between the natural environment and human actions. This is achieved by first presenting a very brief history that begins with broad considerations of the environment (all physical things and conditions surrounding people) and health and culminating in a new public health that stresses, albeit in a somewhat limited fashion early on, ecological thinking. Following this we present a succession of conceptual ecological models of health. Ecological public health models are those that represent individual health as encompassed by larger socio-cultural and physical environment spheres of influence. The inclusion of the natural environment construct in these models has evolved from a broad construct of the physical environment to the inclusion of the natural environment and biosphere, eventually leading us to models that capture complex ecological thinking with natural environment processes assuming a much more prominent position. In our discussion, we present a number of movements attempting to formalize the interconnections between the natural environment and health and conclude that we are now beginning to assemble knowledge of sophisticated ecological interdependencies and apply this knowledge to the conceptualization and study of the relationship between the natural environment and the determinants of human health. 

## 2. The Natural Environment in Increasingly Ecological Conceptions of Health

### 2.1. Public Health and the Environment

The environment has long been considered a determinant of health, but the environment conceived simply as the physical things surrounding people does not take into account the reciprocity between the environment and people. Public health has progressed from considerations of the physical environment as an undynamic external entity to a contemporary acceptance of the ecological paradigm. The ecological paradigm recognizes the relations not only between organisms but also between organisms and their physical surroundings. This has largely been a socio-ecological approach with the organisms in question being humans and the focus on the relationships between humans. Considerations of the other half of ecology, the reciprocity between humans and the environment, especially with respect to the natural environment, have been somewhat limited in the “ecological” public health paradigm.

As McCally states, “at least since the time of Hippocrates’s essay “Air, Water, and Places” humans have been aware of the many connections between health and the Environment” [3]. Duhl and Sanchez [4] present an evolution of public health paradigms, noting that the Ancient Greeks considered the environment critical to health but were largely concerned with finding the “right” environment and not necessarily manipulating the environment to make it “right” for health. Following the Ancient Greeks in an era of nonspecific sanitation, the Romans, with their notable emphasis on civil engineering, were not searching for the “right” environment but instead mastery over it to create an environment conducive to health. Extending through the medieval period and lasting until the germ theory came to prominence in the later 19th century, it was not the control of the physical environment but rather the miasma—or foul, pathogenic, air—that was considered essential to protecting health. The presence of miasma was thought to affect the salubrity—or wellness—of the local environment. “Any major environmental element—land form, water moving and still, climate patterns, vegetation, wind patterns, history of local epidemics—had its role to play in whether or not an observer assessed a site as salubrious or not” [5]. The perceived wellness-enhancing qualities of the natural landscape were a driving force behind 19th century landscape architecture bringing green space into the city [6]. 

The Victorian era public health reforms in the mid to late 1800s have often been lauded as the period when large-scale urban infrastructure was introduced for the explicit purposes of protecting public health. This is a time when urban planning (spatial planning) and public health are essentially inseparable [7] and the disciples of miasma and germ theory were at odds. The reforms of this era are well documented in the literature [8], particularly the literature over the past two decades when there has been a renewed interest in the intersection between urban planning and public health. The miasmists and germ theorists, although for different reasons, were working under a socio-ecological model of public health; the miasmists because they believed that persons of low social station and character were ill because of their increased, and what they considered deserved, exposure to foul air, and the germ theorists because they recognized that social actions, such as poor sanitation, led to exposure to disease-causing pathogens. 

The eventual victory of germ theory proved an advancement in the protection of humans from infectious (communicable) disease, but it did not fare well for an ecological health paradigm. As is often the case if all you have is a hammer, everything looks like a nail [9], the discovery of pathogens led public health into an era of attempting to immunize people against these pathogens, reducing the focus on the environments where pathogens thrived and the means by which humans came into contact with them. Considering the resources devoted to clinical health care today, this is still the dominant paradigm. We see the questioning of the biomedical paradigm when Duhl states “…our old (biomedical) methods of seeking solutions were inadequate and have to be replaced by far more complex and sophisticated perceptions of the interplay between man (people) and his (their) environment” [10]. Decades later, in an attempt to capture this “interplay” and remedy the perceived shortcoming of the biomedical paradigm, we see the idea of salutogenic, or health-promoting, environments taking hold [11]. This type of ecological thinking is reflected in the “new” public health. 

The charter of the new public health was delivered in 1986 at the First International Conference on Health Promotion in Ottawa. In the charter it was declared that “the fundamental conditions and resources for health are peace, shelter, education, food, income, a stable ecosystem, sustainable resources, social justice and equity. Improvement in health requires a secure foundation in these basic prerequisites” [12] (it is in this same year that the WHO Healthy Cities initiative was born, a movement that harkens back to the Victorian era sanitary reforms [8]). This list of conditions specifically credits the reliance on a stable ecosystem and sustainable resources as “fundamental conditions” for health (the term “stable” to denote viable ecosystems is no longer in vogue. It is now understood that a viable ecosystem may constantly be in a state of flux and that its resilience is what is critical to maintain ecosystem functionality). Practitioners of the new (post-1986) public health were seen as needing “…a good grounding in ecology and a vision of how to reconcile the natural and the built environments” [13]. The new public health was charged with revisiting “…all the topics of the old public health—housing, food, water, sanitation, education, occupation, transportation, genetics and microbiology, and medical and social services—and reexamine them with ecological eyes” [13] because “…with many public health issues, several elements must be dealt with in order truly to solve the larger problem” [4]. The Ottawa Charter codified that the natural environment was necessary to deliver many of the fundamental conditions for health. As is outlined in the following section, over the nearly 30 years since the Ottawa Charter, natural environment constructs have been represented with varying degrees of sophistication in ecological models of health. 

### 2.2. The Natural Environment Concept Finds Its Way into Ecological Models of Health

In the era of the new public health, we see a number of ecological models of heath that reflect an appreciation for the complexity of the determinants of health. In early models, we see the physical environment, and then explicitly the natural environment, featured among other determinants but in a non-ecological way that fails to demonstrate interrelationships between the natural and built environment. This does not suggest that the designers of these models were unaware of interrelationships. These models are still very helpful conceptual tools, especially those that accurately include the natural environment and its overarching influence on other determinants. Later models are more accurately ecological and provide us with direction on how protecting the natural environment is essential to supporting public health. 

**Figure 1:**
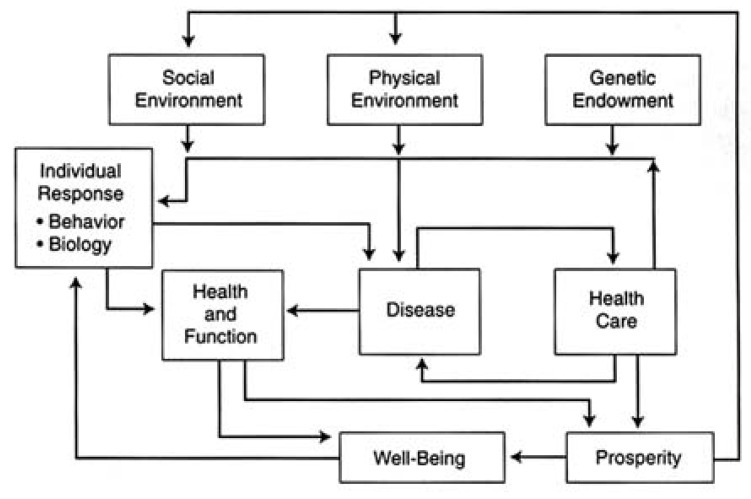
The determinants of health (reprinted from [18] with permission from Elsevier^®^).

**Figure 2:**
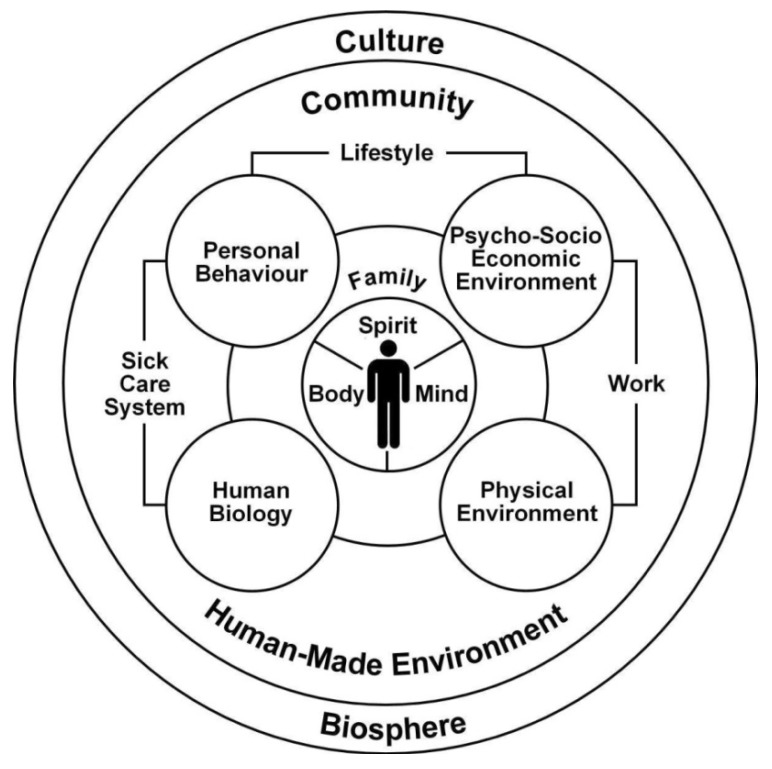
The Mandala of Health (reprinted from [21] with permission from Oxford University Press^®^).

**Figure 3:**
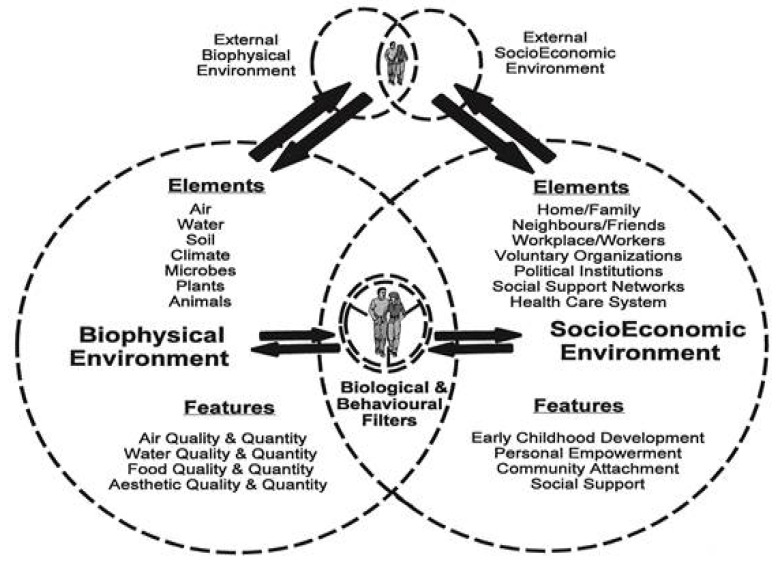
The Butterfly Model of Health (reprinted from [15] with permission from John A. Burns School of Medicine, University of Hawaii).

**Figure 4:**
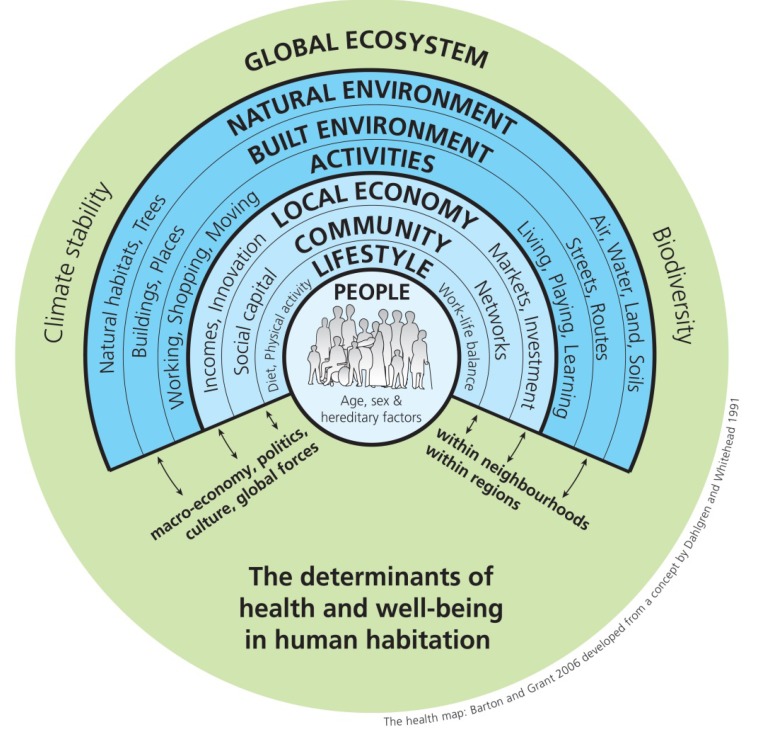
The Health Map [24,25].

**Figure 5:**
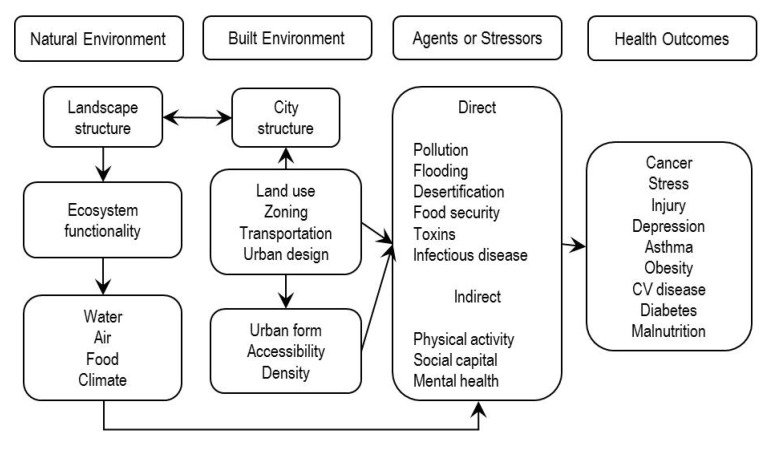
Public Health Ecology (reprinted from [26] with permission from Kristen Ruby).

**Figure 6:**
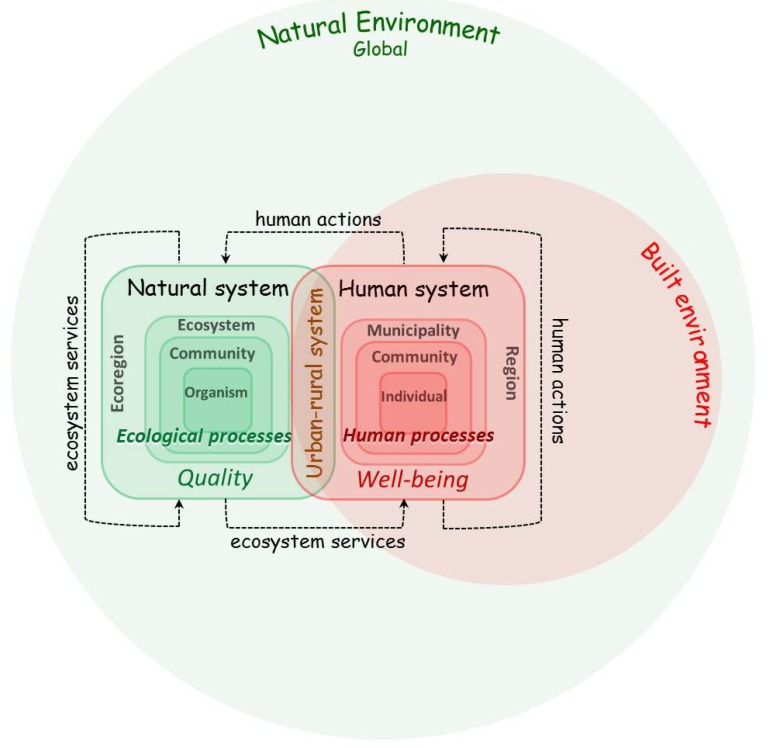
Transformation via Balanced Exchanges Model.

Note: Created by Annet Forkink. Adapted from [31,32,33].

Morris *et al.* [14] and VanLeeuwen *et al.* [15] present a number of iterations of conceptual models which emphasize the sophisticated array of ecological influences on public health (it would be remiss not to also mention the decades of work on ecological models of health promotion done by Lawrence Green. In Green, Richard, and Potvin [16], the evolution of ecological thinking in health promotion is presented along with its pervasiveness in many disciplines that relate to public health). The models found in Morris and VanLeeuwen are represented in Figure 1, Figure 2 and Figure 3 below. Figure 4, Figure 5 and Figure 6 were added by the authors of this paper to extend the progression of ecological thinking. These selected frameworks and models do not, nor were they meant to, represent a fully complete set of the determinants of health. Rather, they offer a way to assemble evidence so that its implications and interdependencies are clear. It is these types of models which have aided our understanding of complex ecological systems and the role of nature in them [17].

The model by Evans and Stoddart [18] (Figure 1) shows the direct influence that the physical environment can have on disease and how the physical environment might operate via the individual response of behavior. It is not clear in this model what is included in the physical environment construct, but it is assumed that the natural environment is part of all of the things and conditions surrounding people. If this assumption is correct, then the natural environment has a direct influence on disease and also indirectly on health and function via individual responses. Health and function influence the prosperity that then influences the physical natural environment. In this sense, with this feedback, the model is ecological, but an appreciation for natural environment can only be assumed. In models to come, the natural environment becomes an overarching force.

Presented in Hancock and Perkins [19] and modified slightly in Hancock [20,21], the Mandala of Health (Figure 2) is among the earliest recognitions of the natural environment and biosphere in a bio-psycho-social-environmental conceptual model (Hancock [22] has also constructed a number of models (The Model of Human Development; The Model of Health and the Community Ecosystem) which squarely recognize the significance of the natural environment in an ecological model of health and the synergy between health and sustainable development). It communicates the human-made (built) environment as subservient to the biosphere that includes the natural environment and processes. With the biosphere encompassing the other constructs in the model, we can assume that there are interactions between them. Here, unlike Figure 1, we can more safely assume that the physical environment construct includes both the built environment and the natural environment.

The Butterfly Model of Health [15] (Figure 3) provides another useful conceptualization regarding the influence of the physical, this time the biophysical, environment on human health. In the tradition of the many evolving models of health presented here and reviewed by VanLeeuwen *et al.*, the Butterfly Model recognizes the crucial role of the socioeconomic environment, but it advances the biophysical (natural) environment as equally significant. 

Humans, and their individual characteristics and behaviors, are nested at the intersection of biophysical and socioeconomic environment. Humans are placed in an ecosystem context with reciprocity between humans and the biophysical environment, recognizing that humans move in and out of a variety of environments which they are both influenced by and have an influence upon. This model more accurately represents the ecological relationship between humans and the environment because it includes not only the influence of the environment on human health but also the influence of humans on the environmental elements necessary to maintain health. In this ecological approach, health becomes a process rather than quantifiable outcome [23] because measures of health status are constantly changing due to dynamic human-nature interactions.

The Health Map in Figure 4 depicts the overarching influence of the global ecosystem and natural environment on the human habitat [24,25] (Barton and Grant, both urban planners, attribute Dahlgren & Whitehead [25] with an antecedent to this figure. The Dahlgren & Whitehead paper was reproduced in 2007 with a slightly revised figure). 

Because the Health Map depicts “human habitation” it has the flexibility to be applied to various scales. The natural environment here could refer to the neighborhood, but also to larger regional systems, both of which ultimately influence and are dependent upon global ecosystems. The built environment and the activity that occurs within it are also ultimately dependent upon larger global forces. Like the Butterfly Model, the human is an agent in the environment, community, and economy that influences health through biological (hereditary) and behavioral lifestyle filters. The advancement in this model is an explicit recognition of climate stability and biodiversity as essential components of the global ecosystem. Climatic variability and diminished biodiversity threaten all other spheres of influence on human health. Although the reciprocity is not explicit in the model, similar to the Mandala of Health, the nesting of all other spheres within the biosphere allows the interpreter of this model to assume that these smaller spheres also influence the biosphere.

A Public Health Ecology model (Figure 5) focuses on the interactions between the natural and built environments and specifically on the health outcomes that result from urban planning decisions that alter the natural environment [26]. 

Much like the Evans and Stoddart model (Figure 1), it explicitly advances the natural landscape as supporting health directly through environmental agents and stressors and indirectly through the behaviors that the environment facilitates or hinders. The presence or absence of elements of the natural environment can directly influence health. In the starkest example of the human dependence on the physical environment, health (and life) would cease without a minimum quantity and quality of water, air, and soil. These natural elements are necessary for health, and the natural environment determines their quality and abundance. Also, the natural environment can influence health through the behaviors that it facilitates or hinders (there has also been an ecologic revolution in the role of the social environment on health behavior [27], but that is not the focus of this paper. The social environment, and rightfully so, continues to receive a great deal of attention in the health literature, the physical natural environment much less so). Environmental barriers such as climate and the distance to and availability of natural resources are contributing factors to the choices people make; choices with health consequences. Unlike Figure 1 and others presented up to this point, Figure 5 shows the reciprocity between the natural and built environments and how alteration to the natural environment influences the ecosystem services necessary to support health. These ecosystem services are dependent upon a sound landscape. It advances the idea that the structure of the landscape—the pattern and connections of conserved land—has an influence on the viability of ecosystems that can be threatened by urban development. 

The examination of the human-nature interactions displayed in all of these models is what is encapsulated in a coupled human and natural systems (CHANS) approach. There has been a swelling of interest in a CHANS research [28,29]. Although not focused specifically on health outcomes, a CHANS approach recognizes that the interrelationship between human and natural systems affect the “…potable water, clean air, nutritious food, raw materials, and medicine” on which health depends [29]. CHANS is an umbrella to the human-environment systems, ecological-economic systems, population-environment systems, or ecological research that overlap one another in various complex ways. “What distinguishes the CHANS approach is an explicit acknowledgement that human and natural systems are coupled via reciprocal interactions…” [30]. In the case of natural environment and public health, we would focus on the social forces responsible for the protection of natural systems and the reciprocal influence these systems have on the health of populations. This is exactly what is described in the Millennium Ecosystem Assessment’s *Ecosystems and Human Well-Being*: *Health Synthesis* [1]. Recognizing that these interactions can in no way be isolated to silos of knowledge, it is communities of researchers like CHANS (but also represented in the list of fields and movements in the Discussion section) that can create the consilience of knowledge between disciplines necessary to deliver health. An example of this CHANS approach—and how it can be adapted to represent an ecological conception of health—is the Transformation via Balanced Exchanges (T-BE) model (Figure 6). 

T-BE presents the exchange of ecosystem services and human actions between the natural and human systems occurring in the built and natural environment contexts. Among the outcomes of these interactions, as evident in the previous models, is human health. In T-BE, the human system, which is the human community within its physical environment, is no longer at the center of the model, but neither is the natural system. Instead, the human system is viewed in the context of the built environment and interacting and overlapping natural systems—the human and natural systems together acting as an integrated part of the larger global natural environment. 

The natural system encompasses ecoregions with ecosystems, communities, and organisms [32]. Ecological processes determine ecosystem functioning and supply of ecosystem services such as food, fiber, and potable water [2,31]. At the interface of the human and natural system are the ecosystems along the urban-rural continuum [34,35]. These ecosystems form the urban-rural system within the overlapping natural and built environments. The human system includes individuals and relationships between individuals at the community and regional (or higher) level. Humans not only depend on the environment via ecosystem services but also impact the environment with their actions. This in turn can have an impact on human health. It is also important to note that ecosystems can also create “disservices” to human health [36]. An example of this disservice is the vectors that carry disease. 

## 3. Discussion

This is an exciting time in public health as there are a number of fields (also movements and disciplines) attempting to formalize the interconnections between health and the natural environment. The fields listed in Table 1 could all be considered as appropriate “homes” for the study of natural environment and health as all employ an ecological approach. These fields draw on others devoted exclusively to either human health or ecological sciences in an attempt to bridge the gaps between human health and ecology.

Missing from Table 1 is the public health specialization of environmental health which would seem an obvious wellspring for research into the ecological health effects of the natural environment and processes. Although there have been calls for the field to move in this direction [37,38] and progress has been made in increased consideration for landscapes and ecosystems—most notably in a focus on sustainability—the toxicological approach to environmental and occupational health still reigns [37,39]. The field charged with protecting the environment for the purpose of public health has been somewhat slow in adopting an ecological perspective that includes the protection of the natural environment.

It is critical to note here that fixing the environment or providing the “right” environment, as the Romans believed, is not a panacea for public health ills. Quite far from an environmentally deterministic view, the ecological paradigm presents the environment amidst, and influenced by, an array of other determinants of health. What is lacking in the current application of the ecological paradigm is the recognition of the full scope of the environment construct which recognizes our evolutionary and still fundamental need for the health-supporting benefits of the natural environment. We have moved beyond the Victorian era belief that incorporating green space into urban environments will cure urban mental and physical ills, but maybe we have moved too far and diminished the fundamental importance of these spaces.

**Table 1:** Summary of fields that connect health and the natural environment.

Field	Description
Conservation Medicine [40]	Connects ecosystem, animal, and human health, but is largely focused on infectious disease transmission exacerbated by human encroachment into animal habitat.
OneHealth [41]	“One Health is the collaborative effort of multiple disciplines-working locally, nationally, and globally to attain optimal health for people, animals and our environment” (p. 13).
EcoHealth [42]	Encompasses Conservation Medicine and OneHealth but also considers equity and development in a socio-ecological systems approach to health.
Human Ecology [38]	The study of human-environment interactions. Health has been considered as an outcome of these interactions.
Health Ecology [43]	Health ecology places health as the core concept in human ecology, the ultimate aim of which “...is the creation and maintenance of healthy people in healthy environments” (p. 17).
Ecotoxicology [44]	The study of the ecological products and mechanisms that have the potential to affect ecosystem functionality and cause illness.
Public Health Ecology [26]	Presents the natural environment as fundamental to health and denotes the reciprocal relationship between the natural and built environment.

The natural environment is absolutely necessary to survive and thrive as a species, and by ignoring the reciprocal maintenance and determinism between humans and the natural environment (putting the burden on the shoulders of individual choice), there is a risk of blaming the victim for health woes. The biomedical model focus on individual immunity—and the provision of therapeutic care when immunity fails—would have us blame poor health on individual failings, either in lifestyle choices or genetic factors. The individual is the centerpiece of an ecological model (Figure 4), but it is clear that the individual can only go so far in choosing among and altering these larger spheres of influence. Victim blaming occurs when we advocate for individual health-promoting behaviors in environments that do not support the proclaimed action. Could we reasonably expect someone to implement proper sanitation in their daily lives in a sprawling informal settlement without the provision of sanitation infrastructure? Could we reasonably expect someone to eat healthier in an environment where healthy food options are non-existent or economically out of reach? It is the same for natural environment. While it is the collection of individual choices that threaten the natural environment, it is the health consequences of a diminished natural environment that are spread to all living things. We blame the victim when we put the burden on individuals for their own diminished health in environments that cannot support health. 

“Development has generated enormous benefits for humanity and improved human wellbeing, but gains through inappropriate practices (e.g., undervaluation and overexploitation of ecosystem services) have also increased risks and impaired numerous ecosystem services essential for human survival and development” [29]. The benefits of the built environment have tended to come at the expense of the natural environment, but there is a precedent for how this might be remedied. It is a contemporary green infrastructure approach that brings to the forefront the natural environment and the ecosystem services on which health depends. Green infrastructure is defined as “an interconnected network of green space that conserves natural ecosystem values and functions and provides associated benefits to human populations” [45]. This is well beyond the inclusion of parks and gardens once considered as oases of respite in bustling cities. An interconnected system of protected national, state and city parks, greenways, and other forms of conserved lands can help optimize ecosystem functioning and ensure the ecosystem services necessary to maintain human system functioning. In doing so, green infrastructure holds an equal, if not more critical, role in supporting health as the grey infrastructure (e.g., water pipes, roads, electrical grid) of the built environment. Without green infrastructure that supports a healthy landscape and ecosystem services, many of the other prerequisites of health are simply undeliverable. 

It is an examination of green infrastructure (not independent of its relation to blue infrastructure (lakes, rivers, oceans) and the atmosphere) for its human health benefits that holds great potential for a consilience between the social, health, and environmental sciences. Using the example of the ebb and flow of collaboration between urban planning and public health, consilience has occurred when an environment issue was deemed the culprit in public health maladies [46]. Green infrastructure could be that critical contemporary issue that makes evident the common goals between environmental sciences and conservation and public health. Working towards the common goal of sustainable development, but focused on a different set of organisms as the benefactors, the environmental and health sciences could potentially have a much greater effect on improving the natural environment if their commonalities were embraced. Our contemporary ecological models of health put forth the notion that the green infrastructure necessary to maintain a viable habitat for non-human organisms is also essential for human health. 

## 4. Conclusions

The natural environment has long been recognized as an important *type* of environment to health, but we are now in a period of unparalleled information revealing the complexity of the natural environment and the processes and services that ecosystems provide to humans. The ecological models of health that include the natural environment present it as fundamental to other spheres and constructs. Despite the overarching influence of the natural environment on human health, it is the other constructs in these models which continue to receive the greatest amount of attention and study. The now decades old warnings of the diminishing state of the environment and ecological threats [47,48] should be pushing public health research and practice to give the natural environment a much more prominent position. After decades of public health’s acceptance of the ecological paradigm, it is time to apply a more accurate ecological conception to the interdependency between the natural environment and human health. These models provide us with some direction on how to achieve this.
